# The Therapeutic Role of Short-Chain Fatty Acids Mediated Very Low-Calorie Ketogenic Diet–Gut Microbiota Relationships in Paediatric Inflammatory Bowel Diseases

**DOI:** 10.3390/nu14194113

**Published:** 2022-10-03

**Authors:** Naser A. Alsharairi

**Affiliations:** Heart, Mind & Body Research Group, Griffith University, Gold Coast, QLD 4222, Australia; naser.alsharairi@gmail.com

**Keywords:** children, short chain fatty acids, very low-calorie ketogenic diet, gut microbiota, inflammatory bowel disease

## Abstract

The very low-calorie ketogenic diet (VLCKD) has been recognized as a promising dietary regimen for the treatment of several diseases. Short-chain fatty acids (SCFAs) produced by anaerobic bacterial fermentation of indigestible dietary fibre in the gut have potential value for their underlying epigenetic role in the treatment of obesity and asthma-related inflammation through mediating the relationships between VLCKD and the infant gut microbiota. However, it is still unclear how VLCKD might influence gut microbiota composition in children, and how SCFAs could play a role in the treatment of inflammatory bowel disease (IBD). To overcome this knowledge gap, this review aims to investigate the role of SCFAs as key epigenetic metabolites that mediate VLCKD–gut microbiota relationships in children, and their therapeutic potential in IBD.

## 1. Introduction

IBD is a distinct chronic, idiopathic, and relapsing disorder classified into two major conditions, including Crohn’s disease (CD) and ulcerative colitis (UC), which cause inflammation in the gastrointestinal tract (GIT). CD affects all parts of the GIT, but is localised most often to the colon and distal ileum, whereas UC affects the colon only [1]. IBD affects not only adults, but also children of all age groups, with higher rates of CD than UC and/or IBD-unclassified (IBDU) reported in most Western countries. In contrast, data from developing countries suggests higher rates of UC than CD [2]. When IBD is detected in children, overlapping histological, radiologic, and clinical features may pose a challenge differentiating between CD and UC [3]. A diagnosis of IBDU occurs when children have features on histological and clinical evaluations that are inconsistent with either CD or UC children [4]. However, it has been shown that IBDU children share similarities of molecular analysis of gene expression and serological features only with UC children two years after the diagnosis of IBDU [5]. Globally, the incidence of IBD in children under 19 years of age at diagnosis increased between 1985 and 2018 as follows: 0.1 to 13.9/100,000 for CD; 0.1 to 10.6/100,000 for UC; and 0.1 to 3.6/100,000 for IBDU [6]. Due to this increase, researchers have been exploring treatment options to manage paediatric IBD. The currently preferred treatments are largely focused on a group of biological agents that have been approved for use in the treatment of CD and UC/IBDU. The anti-tumour necrosis factor (TNF) agents, adalimumab (commercialised as Humira^®^) and infliximab (commercialised as Remicade^®^), have been shown to be effective in reducing moderate-to-severe complications of CD in children, and might be clinically beneficial against UC in children [7]. Vedolizumab (commercialized as Entyvio^®^), a humanised α4β7-integrin antagonist, was also demonstrated by a few retrospective studies to be effective in maintaining remission in children with CD and UC/IBD-U [8]. Recently, etrolizumab, a humanised anti-β7 antibody, has been demonstrated to have treatment efficacy in children diagnosed with moderate-to-severe CD and UC [9].

Although the aetiology of paediatric IBD is not well known, it is thought that key contributing factors, including genetics (e.g., loss of function variants in specific genes) [10] and the environment (e.g., dietary patterns, exposure to antibiotics, air pollution, appendectomy, enteric infections) [11], may lead to the development of disease, which exacerbates inflammatory immune responses. There is also growing interest in gut microbiota as another potential factor contributing to IBD pathogenesis. The colonization of the gut with diverse microbes is thought to occur during delivery and immediately after birth, and is influenced by multiple factors, including maternal gut microbiota, nutrition, antibiotic exposure, mode of feeding/delivery, and body mass index (BMI) [12]. Immune system development in early life may interact with gut microbiota composition, and the depletion of beneficial microbes may increase the risk of inflammatory diseases [12]. The gut microbiota has maintained a symbiotic relationship with its host through a range of functions, such as facilitating immune system development, protection from pathogenic bacteria, strengthening the integrity of the digestive tract, and production of beneficial metabolites such as SCFAs [13]. Butyrate, propionate, and acetate are the main fermentation-derived SCFA metabolites from indigestible complex carbohydrate (CHO) produced by gut microbes belonging to the phylum, *Firmicutes*, through a range of cross-feeding mechanisms/microbial metabolic pathways [14]. The metabolic cross-feeding of lactate, an intermediary metabolite formed by *Bifidobacterium* and lactic acid bacteria (LAB), can enhance the production of butyrate [15]. Although the gut microbiota composition of children with IBD differed from that of healthy children in several studies, it is consistently characterised by reduced abundances of SCFA-producing bacteria considered as ‘healthy microbiota’. The gut microbiota of children with CD and/or UC showed decreased genera belonging to the phyla, *Actinobacteria* (*Bifidobacterium*), *Firmicutes* (*Lactobacillus*, *Blautia, Ruminococcus*, *Faecalibacterium prausnitzii*, *Roseburia*), and *Bacteroidetes* (*Bacteroides*). However, the genera, *Escherichia*, *Actinobacillus*, *Granulicatella*, *Enterococcus*, and *Streptococcus*, were observed to have increased [16,17].

Diet has proven to be a major factor influencing gut microbiota composition, and, thus, is thought to be a significant environmental trigger in IBD. For example, consuming a high-fat diet and/or high-sugar diet could result in gut microbiota dysbiosis, characterised by reduced SCFA-producing bacteria, thereby dysregulating gut immune homeostasis and increasing susceptibility to gut inflammation, all of which contribute to IBD risk [18]. The results generated from intervention studies in adult IBD patients suggest that dietary fibre and prebiotics increase SCFA production and the abundance of bacteria they produced, thereby reducing gut inflammation and IBD risk [19]. It has been recently shown that higher intakes of prebiotics and other beneficial foods, including fruits, vegetables, honey, oats, legumes, omega 3 fatty acids, and lean animal protein, increase SCFA-producing bacterial taxa abundances and reduce levels of inflammatory cytokines (e.g., interleukins IL-6/IL-8 and TNF-α) in adult patients with CD and UC [20]. The CD exclusion diet combined with partial enteral nutrition or the exclusive enteral nutrition (EEN), provided in a liquid, low-saturated-fat/heme/taurine and high-protein polymeric formula, are found to be effective nutritional therapies in inducing remission in children with CD [21,22,23,24,25]. However, the introduction of EEN therapy was found to decrease SCFA levels (including butyrate) and the α-diversity of SCFA-producing bacteria, with a lower abundance of *Bifidobacterium*, *Bacteroides*, *Ruminicoccus*, *F.prausnitzii*, and *Blautia* [21,26,27,28], which are thought to be associated with reduced gut inflammation in CD. Thus, there is a need to explore food-based, diet-induced microbial changes to reduce gut inflammation in IBD. Treatment with the specific carbohydrate diet (SCD) that includes corn, wheat, food additives, and milk, or SCD with rice and oats, have been shown to increase the relative abundance of *Blautia* and *F.prausnitzii* in children with CD [29]. Adherence to the Mediterranean diet based on a high intake of fruits, vegetables, seafoods, olive oil, legumes, and nuts, has been associated with increased gut SCFA-producing bacterial diversity, with an increased abundance of *F.prausnitzii* in children [30], and has also demonstrated a significant decrease in inflammatory cytokines (IL-12, IL-13, IL-17, and TNF-α) in children with CD and UC [31].

There has been increased interest in the standard ketogenic diet (KD); namely, the very low-calorie ketogenic diet (VLCKD) as an effective restricted dietary pattern influencing SCFA-producing bacteria, thereby modifying inflammation-associated disease risk in early life [32,33]. There is little evidence to support the health benefits that the VLCKD has in paediatric IBD. Only one case report has revealed that Palaeolithic KD improves CD symptoms and progression in children [34]. It is hypothesized that the VLCKD influences SCFA-producing bacteria and reduces gut inflammation in IBD. However, the mechanism by which SCFAs could mediate VLCKD–gut microbiota relationships and the therapeutic implications for reducing IBD are largely unknown. Thus, this review highlights the potential role of SCFAs in the epigenetic mechanism underlying these effects.

## 2. Methods

A literature search of the PubMed/Medline database was carried out to identify in vitro/human studies published in English over the last 20 years using the following search keywords: children, IBD, intestinal epithelial cells (IECs), intestinal inflammation, inflammatory markers, VLCKD/KD, SCFA, KBs, gut microbiota, and epigenetic. Experimental studies, observational studies, randomised controlled trials (RCTs), and reviews were included for the purposes of this review. In vivo studies or studies of animal models were excluded from the review.

## 3. Epigenetics of Paediatric IBD

Epigenetics has revealed a potential mechanism that may explain how environmental triggers and genetic susceptibility interact in IBD [35]. Evidence from many studies suggests that epigenetic modification, in particular DNA methylation, which exists at cytosines in the cytosine–guanine (CpG) dinucleotide context, plays a key role in paediatric IBD phenotypes, as it is considered a key regulatory mechanism of gene expression in response to environmental cues, without modifying the primary nucleotide sequence [36,37]. However, the little evidence that exists examining histone methylation suggests that this modification takes place in the intestine of paediatric IBD. In CD children, genes exhibiting decreased histone H3-lysine 4 trimethylation (H3K4me3) signatures are found to be associated with the severity of inflammation in IECs [38]. Ileal IECs play a significant role in integrating commensal microbiota-derived cues to regulate immune homeostasis and gene expression. Genes characterized by increased H3K4me3 levels (e.g., *DUOX2*, *NOS2*) in IECs from CD children in response to commensal microbiota are enriched in several pathways, including the regulation of reactive oxygen species (ROS), G alpha signalling, digestive system development, and nitric oxide (NO) biosynthesis, suggesting that commensal microbiota may modify histone alterations that reflect intestinal inflammation in CD [38]. Large-scale, genome-wide studies have revealed significant mucosal DNA methylation changes in IBD-associated genes in children. For example, a systematic meta-analysis of 84 genetic studies identified specific genetic variants (rs11209026, rs7517847, rs12521868, rs26313667, rs1800629, rs2241880, rs2066847, rs2066844, and rs2066844) in differential DNA methylated genes (*NOD2*, *IL23R*, *ATG16L1*, *IBD5*, and *TNF-α*) known to cause CD and UC [36]. A study that assessed DNA methylation profiles of the colonic mucosa found that 182 CD and 3365 UC susceptible genes (including *STAT3*, *SLPI*, *ITGB2*, *SAA1*, *IFITM1*, and *S100A9*) were associated with differentially methylated regions (DMRs) [39]. Another study detected a number of UC-associated changes in DNA methylation at nine CpG sites located in the *TRIM39*-*RPP21* gene [40]. In respect to the correlation between the colonic mucosal DNA methylation of paediatric IBD and microbiome changes, a study showed that *SLC9A3*, a gene with decreasing methylation UC-specific DMR, was associated with a reduced abundance of *Bacteroides* [41].

## 4. Role of Inflammatory Biomarkers in Paediatric IBD

Loss-of-function mutations in G-protein coupled receptors (GPCRs) may result in reduced ligand-binding affinity to many chemokines/*cytokines*, local mediators, and neurotransmitters during childhood development [42], which may lead to an increased risk of IBD. Naïve CD4^+^ T cells differ according to cytokine-producing T-helper (Th) subsets, including Th1 (interferon-gamma, IFN-γ, TNF-α), Th2 (IL-4, IL-5, IL-6, IL-13), and Th17 (IL-17) cells, which are implicated in the dysregulation of colonic mucosa in IBD patients [43,44]. Paediatric CD patients were shown to have a mixed Th1/Th2/Th17 cytokine profile, with increased serum levels of IL-1β, IFN-γ, IL-6, TNF-α, C-X-C motif chemokine ligand 10 (CXCL10), and IL-17A observed in both the ileum and colon [45,46]. Increased serum IL-4 and IL-6 levels were detected in the intestinal mucosa of paediatric UC patients, in which the GATA binding protein 3 (GATA3) and signal transducer and activator of transcription-4 (STAT4) signalling molecules were involved [47]. Compared with paediatric CD patients, significantly more mRNAs related to IL5, IL-13, IL-23, and IL17A cytokines were observed in the rectal mucosa of UC patients [48]. Serum levels of IL-6 were found to be higher in the ileum of paediatric CD patients than those of healthy children, whereas the serum levels of IL-22 and IL-17A were higher in UC patients than in CD patients [49]. Although Foxp3^+^T_reg_ cells are found in higher density in the inflamed mucosa of paediatric IBD patients, they maintain immune homeostasis [49,50]. A higher density of Foxp3^+^ cells in the ileum of untreated paediatric CD patients compared with adult patients may be attributed to the disparate pattern of CD phenotypic expression [51]. In a previous in vitro experiment, serum levels of IL-17 and TNF-α were increased in the peripheral blood of paediatric patients with CD and UC. Patients displayed a decreased expression of Foxp3^+^, CD4^+^, and CD25^+^, and an increased percentage of Th17 cells. Myeloid dendritic cells (mDCs) and plasmacytoid DCs (pDCS) expressing CD200, a type I transmembrane glycoprotein, were found to be elevated, and significantly associated with Th17, but negatively associated with regulatory T cells (T_regs)_. On the contrary, the expression of the CD200 receptor, CD200R1, on mDCs was found to be reduced and negatively associated with Th17 [52]. The mRNA expression of pro-inflammatory cytokines IL-6 and IL-23 expressed at high levels in the colonic mucosa of paediatric patients with CD and UC is found to be associated with a higher frequency of CD4^+^ IL-17a^+^ and a lower frequency of CD4^+^Foxp3^+^T_regs_ [53]. An experimental study demonstrated that treatment with infliximab, a chimeric monoclonal antibody, does not inhibit the production of TNF-α, but also hampers expansion of FOXP3^+^ T_regs_ in the colonic mucosa of paediatric CD patients [54]. Another recent experimental study reported that paediatric patients with active CD and UC, though having a high expression of CTLA-4 in FOXP3^+^ T_regs_ in peripheral blood during pharmacological (aminosalicylates or azathioprine) therapy, showed a significant decrease of CD4^+^Foxp3^+^T_regs_ levels after therapy compared to the same patients and healthy children at disease onset [55].

In a human IBD model, which is characterised by increased histone deacetylases (HDACs), nuclear factor-κB (NF-κB), nuclear factor kappa-B kinase β (IKKβ), TNF-α, *NOD2*, and toll-like receptor (TLR) upregulation have been reported to occur in the inflamed IECs, resulting in high pro-inflammatory cytokine expression levels [56]. HDAC1 has been shown to be implicated in several diseases, in which it takes off the acetyl group from lysine residues of histone/non-histone proteins via acetyltransferases (HATs), which results in DNA inaccessibility, gene transcription repression, and chromatin compression [57]. The acetylation of histone H3 lysine 27, which is identified in regions with several risk loci for IBD [58], has been found to be downregulated in the inflamed mucosa of UC patients, resulting in high IL-6 mRNA levels [59]. HDAC1 induces an inflammatory response in the colon epithelium of UC patients by activating NF-κB, and reducing histone H3 acetylation and tight-junction protein, zonula occludens 1 (ZO-1), expression [60]. Loss-of-function *NOD2* gene mutations enhance NF-κB activation, which, in turn, bind to the promoters of pro-inflammatory cytokines in paediatric CD patients [61]. This suggests that cytokines with pro-inflammatory effects could play a key role in the pathogenesis of paediatric IBD.

## 5. The VLCKD and Its Relationship to Gut Microbiota

The VLCKD is characterised by a high proportion of fat (70%), adequate protein (20%), and a low proportion of carbohydrates (CHO—10%, <50 g per day) [62]. This diet favours animal and/or plant-based sources of dietary fibre, protein, monounsaturated fatty acids (MUFA), polyunsaturated fatty acids (PUFA), and saturated fatty acids (SAT) (e.g., non-starchy vegetables, chicken, lamb, beef, eggs, cream, cheese, butter, olive oil) [63]. The VLCKD restricting CHO to either simple or complex CHO remains an active area for further investigations. Unlike sugars or simple starches, the dietary fibre, including both non-starch polysaccharide and resistant oligosaccharides-based foods, metabolised by the human colonic microbiota generate SCFAs [64,65,66,67,68,69], which play a significant role in regulating inflammatory immune responses [65,67]. Dietary fibre can also be considered as prebiotic-like galacto-oligosaccharides, which stimulates the growth of the gut microbiota and improves host health [66]. The daily fat intake should be high enough at around 70% of total calories to replace CHO intake and maintain ketosis. The daily protein intake should be kept moderate, at around 20% of total calories, because low-CHO–high-protein diets may not induce ketosis [62].

The VLCKD would be beneficial to achieve nutritional ketosis, a state known for its anti-inflammatory effects [32,33], which is characterized by increased blood levels of acetoacetate (ACA) and β-hydroxybutyrate (βOHB), the two main ketone bodies (KBs) produced in the liver and used as secondary energy sources when CHO stores are depleted [70]. βOHB has a structure and role in regulating gut homeostasis and gene expression through epigenetic modifications similar to butyrate, both of which may have therapeutic roles in treating inflammation-related diseases, such as obesity and asthma, in children [32,33]. During adherence to a VLCKD, hepatic glycogen stores are decreased, and FFAs are produced as an alternative energy source from adipocytes and regulated by insulin and glucagon. FFAs are then converted by β-oxidation to acetyl-coenzyme A (acetyl-CoA) in a process regulated by several enzymes that induce βOHB formation [71]. Butyrate serves synergistically with βOHB on inducing ketosis, in which it acts as a ligand to enhance receptors that the βOHB will also work on. Butyrate promotes fibroblast growth factor 21 (FGF21) in the liver, which enhances fatty acid β-oxidation for the production of βOHB, which can induce ketosis [72]. βOHB administration on human colonic microbiota models has been found to increase butyrogenesis and butyrate production [73].

The role of VLCKD in modulating/influencing the gut microbiota composition in paediatric IBD has been not investigated yet. The VLCKD has been shown to affect the gut microbiota of children by influencing the growth of SCFA-producing commensal microbes (e.g., *Bifidobacterium*, *Lactobacillus*, *Bacteroides*, *Faecalibacterium*, *Clostridium*, and *Ruminococcus*) in which epigenetic changes are involved [32,33]. The VLCKD exhibits an increase in circulating levels of βOHB, and induces epigenetic changes in the gut microbiota, suggesting that the anti-inflammatory effects of VLCKD in reducing IBD in children may be due to an increase in anti-inflammatory βOHB through epigenetic mechanisms by which SCFAs-producing bacteria could reduce the pro-inflammatory cytokines and chemokines in IBD children.

## 6. SCFAs as Epigenetic Modifiers in IBD

Diet–gene interactions play a key role in CD and UC pathogenesis, and this is why epigenetic modification of DNA/histone methylation could provide new insights outside the context of genetics [74]. Diet–microbiota interactions mediate epigenetics via modulating epigenetic mechanisms and establishing IBD-associated dysbiosis, which can result in an increased risk of CD and UC [74,75]. SCFAs produced by anaerobic gut microbial fermentation of dietary fibre-rich substrates may act as epigenetic mechanisms in regulating IBD-related inflammation through inhibiting cytokine production (e.g., IL-17), which plays a key role in the pathogenesis of IBD [76].

SCFAs, and butyrate and propionate in particular, regulate intestinal homeostasis and immune responses, not only via the inhibition of HDACs, but also by the activation of GPRs, including GPR41 (free fatty acid 3/receptor 3; FFA3/FFAR3) and GPR43 (FFA2/FFAR2) in macrophages and DCs, which enhance the differentiation of T_regs_, characterised as the CD4^+^CD25+ Foxp3^+^ phenotype, and anti-inflammatory activities in colonic mucosa [76,77,78]. Modifications in DNA methylation in response to commensal microbiota and their metabolites (e.g., SCFA) may contribute to the maintenance of paediatric IBD via a variety of mechanisms [37]. UC patients with active disease display a significant decrease in total SCFAs, butyrate, propionate, and acetate concentrations, whereas patients in remission had higher butyrate concentrations compared with healthy counterparts [79]. Butyrate metabolism is the main down-regulated pathway associated with adult and paediatric UC, which reduces the gene expression levels of *BDH2*, *ACSM3*, *EHHADH*, and *HMGCS2* in the intestinal mucosa of UC patients [80]. Butyrate has been shown to regulate mitochondrial gene expression associated with dual oxidase 2 (*DUOX2*) genotype-induced ROS, particularly the DUOX2 loss-of-function haplotype, which is implicated in paediatric CD ileal strictures [81]. Butyrate and propionate exert anti-inflammatory effects on the human intestinal enteroid (HIE)-derived monolayer by modulating adherent-invasive *Escherichia coli* (AIEC) virulence gene expression, implicated in the invasion of intestinal cells, while enhancing the integrity of the epithelial barrier, and reducing gut inflammation through downregulating cytokines TNF-α, IL-6, IL-8, and CXCL family gene expression [82]. A previous experimental study has shown that butyrate inhibits TNFα release and lipopolysaccharide (LPS)-induced IL-6 and IL-1β mRNA expression in the inflamed mucosa of adult CD patients via downregulation of the NF-κB pathway [83]. In an in vitro study relevant to IBD, butyrate and propionate were found to be more effective than acetate in inhibiting LPS-induced TNFα production from neutrophils and TNFα-mediated NF-κB activation in the human colon adenocarcinoma cell line [84]. The expression of SCFA receptor GPR43 identified on intestinal endocrine L-cells has been shown to be reduced in CD patients fed with high-fat/sugar diets [85]. It has been shown that GPR43 mediates the therapeutic activity of butyrate in IBD, in which butyrate exhibits a high potency barrier, enhancing activity in IECs, and inhibiting LPS-induced TNFα, IL-6, IL-1β and IFN-γ, IL-17 release in human peripheral blood mononuclear cells (PBMCs), which were all found to play a significant role in IBD development in children [86]. Butyrate and/or propionate play a significant role in the maintenance of human colon IECs by suppressing HDACs and NF-κB signalling in response to TLR activation [87,88]. In human colon IECs, butyrate suppresses HDACs, thereby promoting intestinal epithelial barrier function through hypoxia inducible factor-1 (HIF-1) and STAT3 activation, which regulate the integrity of epithelial tight junctions, and inhibiting LPS-induced NF-κB activation, which, in turn, decreases pro-inflammatory cytokines (e.g., IL-6, TNF-α, iNOS), while increasing anti-inflammatory cytokine (e.g., IL-10) expression [89]. This suggests that SCFAs, particularly butyrate, may serve as a key epigenetic metabolite that exhibits anti-inflammatory effects in IECs, providing a potential therapeutic role for paediatric IBD treatment via the mechanisms for epigenetic regulation. 

## 7. KBs as Epigenetic Modifiers in IBD

βOHB administration on the colonic mucosa of IBD patients has been shown to increase levels of IL-4Ra- and IL-10-induced M2 macrophage polarisation through activation of the STAT6 signalling pathway [90]. βOHB increases histone lysine β-hydroxybutyrylation (Kbhb) in human embryonic kidney 293 (HEK293) cells, which is a type of histone posttranslational modification responsible for regulating gene expression and chromatin structure [91]. In vitro, βOHB inhibits HDAC1 and enhances histone H3 acetylation in macrophages through binding to GPCRs, which results in increased FOXP3^+^ gene expression and inhibited NOD-like receptor pyrin-domain containing-3 (NLRP3) inflammasome activation, which could, in turn, inhibit the expression of pro-inflammatory cytokines involved in IBD [71]. In human models, NLRP3 inflammasome, which is implicated in paediatric IBD [92], has been shown to be inhibited by βOHB, resulting in decreased pro-inflammatory cytokines IL-1β and IL-18 production in LPS-activated human monocytes with significantly increased histone H3 acetylation in macrophages [93]. HDAC1 is the major Kbhb deacylase in vitro, in which it reduces Kbhb levels in HEK293 cells [94]. In these cells, Kbhb levels on H3K4 residue, which are associated with the severity of inflammation in IECs in children [38], have been found to increase upon treatment with βOHB [94], suggesting that βOHB may have therapeutic potential in paediatric IBD, manifested in regulating gene expression profiles by increasing Kbhb levels in HEK293 cells. It can be suggested that βOHB as a potential epigenetic modifier exerts anti-inflammatory effects in IECs through its ability to attenuate intestinal inflammation and reduce damaged intestinal tissues in paediatric IBD.

## 8. Gut Microbiota-Derived SCFAs as Therapeutic Potential Agents in Paediatric IBD

This section aims to summarise the evidence supporting the therapeutic potential of gut microbiota-derived SCFAs in paediatric IBD.

### 8.1. Bifidobacterium and Lactobacillus spp.

Probiotic strains of SCFA-producing *Bifidobacterium* and *Lactobacillus* have been demonstrated to reduce IBD in human models by improving the intestinal epithelial barrier integrity and regulating the host immune response [95,96,97]. The most commonly used probiotics in RCT studies for the treatment of IBD were *L.plantarum*, *L.acidophilus*, *L.actis*, *L.reuteri*, *L.delbrueckii* subsp. *Bulgaricus*, *B.breve*, *B.longum*, *B.infantis*, and *B.bifidum* [98]. The bacterial abundance of *Bifidobacterium* and *Lactobacillus* spp. was reported to be reduced in paediatric IBD patients [16,17]. Several RCTs/experimental studies demonstrated the therapeutic potential of *Bifidobacterium* and *Lactobacillus* strains in IBD. The results of in vitro experiments showed that several strains belonging to the species, *B.longum* and *L.plantarum*, exert anti-inflammatory effects on IECs, as indicated by increased anti-inflammatory cytokine IL-10 and reduced TNF-α, NF-κB, IFN-γ, and pro-inflammatory cytokines (IL-2, IL-4, IL-6, IL-8, IL-17, IL-1β) production, which are involved in the pathogenesis of paediatric IBD [99,100]. Evidence from an in vitro experiment showed that probiotic supplementation with the *B.longum* strain BL05 in combination with the *B.lactis* BL04 and *L.rhamnosus* LR32 strains were able to modulate the immune inflammatory response of monocyte-derived M1 macrophage by increasing cytokine IL-10 and reducing the production of cytokines IL-6 and IL-1β [101]. Another recent experimental study showed that a mixture of four probiotic strains (including *L.acidophilus* LA1, *L.paracasei* 101/37, *B.animalis* spp. Lactis Bi1, and *B.breve* Bbr8) inhibit IL-8, IL-23, and IL-1β cytokine production in monocyte-derived dendritic cells (MoDC) from UC patients [102]. Treatment with *B.bifidum* NCC189 and S17, *B.longum* NCC2705, and *B.lactis* NCC362 has a potentially inhibitory effect on LPS-induced NF-κB activation and mRNA expression of TNF-α, IL-8, and cyclooxygenase 2 (Cox-2) in the IECs of IBD patients [103]. The administration of different probiotic strains of *B.bifidum* has been shown to enhance colonic acetate production in vitro, which exerts anti-inflammatory effects by inhibiting TNF-α expression [104]. *B.bifidum* strains (BbrY and BbiY) have been found to enhance cytokine IL-10 production and reduce cytokine IL-8 production in the PBMCs of UC patients [105]. Probiotic *B.infantis* 35624 and *L.salivarius* UCC118 strains exert anti-inflammatory effects on HT-29 human IEC by inhibiting *Salmonella typhimurium* (*S.typhimurium*)-induced TNF-α, NF-κB p65, cytokine IL-8 production, and increasing cytokine IL-10 production [106]. The treatment of HT-29 human IEC with *B.bifidum* strains (BGN4-SK and BGN4-pBESIL10) inhibits TNF-α and cytokine IL-8 production [107]. Treatment with *L.brevis*, *L.pentosus*, and *L.curvatus* reduces *Salmonella*-induced-IL-1β, IL-6, IL-8 mRNA levels and the p-IκB-α level, and increases cytokine IL-10 production and zonula occludens-1 (ZO-1)-mediated tight-junction integrity in HT-29 human IEC via the inhibition of the NF-κB pathway [108]. *L.plantarum* LM17 and *L.rhamnosus* LM07 strains exert anti-inflammatory activity in TNF-α-induced HT-29 human IEC by reducing cytokine IL-8 production [109]. *L.kefiri* strain CIDCA 8348 reduces TNF-α, IFN-γ, cytokines IL-6, IL-13 production, and increases cytokine IL-10 production and CD4^+^ FOXP3^+^ T cell expression in the inflamed mucosa of IBD patients [110]. Treatment with *B.adolescentis* ATCC 15703, *B.longum* ATCC 15697, and *B.breve* (ATCC 15700) strains has shown anti-inflammatory effects on macrophages in vitro by inhibiting LPS-induced TNF-α and IL-1β mRNA, while increasing IL-10 mRNA levels [111]. The administration of the *B.longum* strain, CECT 734, to children with newly diagnosed CD resulted in reduced serum TNF-α expression and peripheral CD3^+^ T lymphocytes [112]. The administration of the *L.reuteri* strain, ATCC 55730, to UC children has been shown to increase cytokine IL-10, and to reduce TNF-α and cytokines IL-8 and IL-1β production [113]. Taken together, these findings suggest that probiotic *Bifidobacterium* and *Lactobacillus* strains may have a therapeutic role in reducing paediatric IBD by their ability to exert anti-inflammatory effects in inhibiting inflammatory markers.

### 8.2. Bacteroides spp.

*B.vulgatus* and *B.thetaiotaomicron* are commensal butyrate-producing bacteria [114,115], with anti-inflammatory activity which may have a potential regulatory role in reducing inflammation in the context of paediatric IBD. The relative abundance of both bacteria has been found to be decreased in the gut mucosa-associated microflora of paediatric IBD [116]. Probiotic dietary supplementation with *B.thetaiotaomicron* is regarded as safe and tolerable in paediatric CD patients [117]. In one experimental study, *B.vulgatus*, compared to *Escherichia coli* (*E.coli*), failed to increase TNF-α-induced IL-8 production, as well as NF-κB transcriptional activity activation, in HT-29 human IEC in the presence of CD- and UC-derived PBMC [118]. In another recent experimental study, the isolated LPS*_Bv_* from *B.vulgatus*, compared to that of *E.coli* LPS, exerted immunomodulatory effects as indicated by their ability to inhibit cytokines IL-6 and IL-8, NF-κB, TNF-α, and CXCL-8 production [119]. *B.thetaiotaomicron* produces nanosized outer membrane vesicles (OMVs), which serve a mediating role in microbe–host immune interactions, and exert anti-inflammatory activity in ILCs via increasing cytokine IL-10 production by colonic DC [120]. *B.thetaiotaomicron* attenuates intestinal inflammation via mechanisms related to the modulation of tryptophan metabolism in inflamed intestinal tissues. Particularly, *B.thetaiotaomicron* enhances the differentiation of anti-inflammatory Th1/Th17 cells by modulating CpG within the Foxp3^+^ promoter, thereby inducing T_regs_ differentiation [121]. This suggests that *B.thetaiotaomicron* and *B.vulgatus* may reduce intestinal inflammation in paediatric IBD patients by inhibiting inflammatory gene expression in IECs.

Although some strains of *B.fragilis* (e.g., enterotoxigenic *B.fragilis*) are enteric pathogens [122], other commensal strains detected on the inflamed colonic mucosa of CD and UC paediatric patients produce surface immunomodulatory capsular polysaccharide A (PSA) [123], which has been found in vitro to increase cytokine IL-10 production, inhibit LPS-induced monocyte TNF-α, and induce CD39^+^ Foxp3^+^ T_regs_ in a DC-dependent manner [124]. It has been shown that *B.fragilis* with a PSA on foetal enterocytes inhibits IL-1β-induced IL-8 production by binding to the TLR2 receptor on CD4 lymphocytes to promote the proliferation of FOXP3^+^ T_regs_ cells, resulting in increased IL-10 production [125]. *B.fragilis* during CD exacerbation produces virulent genes, such as *bft* (fragilysin), but this was found to induce IEC resistance rather than disrupting the barrier [126]. This suggests that *B.fragilis* may effectively reduce intestinal inflammation in paediatric IBD, but further studies are needed to evaluate its anti-inflammatory effects on IECs.

### 8.3. Faecalibacterium prausnitzii

*F.praunsitzii* is a commensal butyrate-producing bacterium which has a potential role in gut homeostasis and in promoting anti-inflammatory effects on human IECs [127]. The gut microbiota composition in paediatric IBD is characterised by low *F.praunsitzii* abundance [128,129]. Butyrate produced by *F.praunsitzii* exerts anti-inflammatory effects on in vitro PBMC and DC by reducing IFN-γ and cytokine IL-12 production, increasing cytokine IL-10 production [130,131], and inhibiting TNF-α and NF-κB activation in HT-29 human IEC [132]. *F.prausnitzii* was found to improve the barrier permeability of Caco-2 monolayers in vitro by increasing cytokine IL-10 production, while reducing the production of NF-κB and TNF-α, along with inflammatory cytokines such as IL-1β and IκBKβ [133]. *F.prausnitzii* induces human monocyte-derived and myeloid DC to prime CD4 T cells producing IL-10, induces Foxp3^+^ expression, and inhibits LPS-induced TNF-α and cytokine IL-12 production through modulating the TLR2/6 and c-Jun N-terminal kinase (JNK) signalling pathway [134]. Thus, *F.praunsitzii* may be involved in reducing inflammatory markers in IECs and producing anti-inflammatory effects in paediatric IBD.

### 8.4. Roseburia intestinalis

Gut microbiota dysbiosis in paediatric CD/UC patients is well known for its reduced abundance of butyrate-producing *Roseburia* spp. [17,135,136]. A significant depletion of *Roseburia* in IBD is likely associated with low levels of *R.intestinalis*, which is considered the most abundant species that has the ability to maintain gut homeostasis and induce anti-inflammatory responses through mechanisms for IBD regulation [137]. The relative abundance of *R.intestinalis* has been recently found to be decreased in paediatric CD and UC patients [138]. The *R.intestinalis* strain, DSM 14610, has been shown, in vitro, to breakdown oligofructose to produce butyrate, only by the presence of acetate produced by the *B.longum* strain, BB536, in the growth medium, suggesting a cross-feeding between *R.intestinalis* and *B.longum* [139]. In vitro studies have also shown that *R.intestinalis* DSM 14610 has the ability to produce butyrate in the presence of acetate produced by bifidobacterial strains [140]. Given the fact that *R.intestinals* is identified as a butyrate producer, strains of such species may be involved in inflammatory immune response regulation, and thereby a potential probiotic in the treatment of paediatric IBD. *R. intestinalis* was able to suppress gut inflammation by inhibiting LPS-induced IL-17 secretion and promoting T_reg_ differentiation in colitis [141]. The *R.intestinalis* strain, DSM 14610, stimulates TGF-β mRNA secretion and promotes colonic T_reg_ in LPS induced Caco-2 cells in CD patients [142]. The *R.intestinalis* strain, DSM 14610, alone or in combination with the *F.prausnitzii* strain, A2-165 (DSM 17677), and *Bacteroides faecis* (*B.faecis*) strain, DSM 24798, exerts beneficial anti-inflammatory effects on Caco-2 and HT29 cells in vitro by a significantly suppressed LPS cocktail of (IL-1β, TNF-α, IFN-*γ*)-induced claudin-2 protein expression [143]. Further in vitro studies are needed to assess the anti-inflammatory effects of *R.intestinalis* and the mechanisms behind its beneficial action in reducing paediatric IBD.

**Figure 1:**
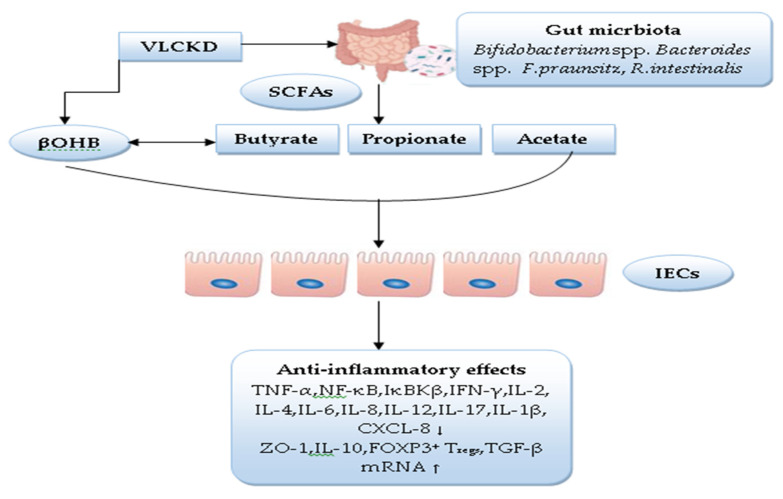
Role of SCFAs as a mediator in paediatric IBD therapy. (↓) decrease, (↑) increase.

Figure 1 summarizes the mediating role of SCFAs in paediatric IBD therapy.

## 9. Conclusions

Diet and other environmental factors may play a crucial role in establishing dysbiosis of the gut microbiota involved in the pathogenesis of IBD. SCFAs are considered the key epigenetic metabolites that mediate the relationships between the VLCKD and gut microbiota in children. SCFAs and βOHB may have the ability to induce epigenetic modifications in the inflamed colonic mucosa of paediatric IBD. Butyrate acts synergistically with βOHB to reduce intestinal inflammation in paediatric IBD and inhibit HDAC, reduce inflammatory cytokine production, and increase histone H3 acetylation in macrophages. SCFA-producing bacteria appear to have a significant role in promoting gut barrier integrity and reducing the production of inflammatory cytokines involved in paediatric IBD caused by a dysregulation of the colonic mucosa. The adherence to the VLCKD may result in increased SCFA-producing bacteria in paediatric IBD, including *Bifidobacterium* spp., *Lactobacillus* spp., *Bacteroides* spp., *F.praunsitzii*, and *R.intestinalis.* SCFA-producing probiotic *Bifidobacterium* and *Lactobacillus* strains may have beneficial effects in modulating immune responses in the inflamed mucosa of paediatric IBD patients. Butyrate-producing bacteria, including *Bacteroides* spp., *F.praunsitzii*, and *R.intestinalis*, may be a potential treatment for paediatric IBD due to their ability to reduce inflammation in IECs. Given that the VLCKD influences the gut SCFA-producing bacteria in children, it may have a potential role in inducing remission and mitigating inflammation in IBD, but further studies are needed to evaluate whether changes in the gut microbiota-producing SCFAs are associated with specific inflammation markers before, during, and after treatment with the VLCKD. Further studies of the VLCKD to assess its safety for the treatment in paediatric IBD patients are also needed.

## Data Availability

Not applicable.

## References

[1] Fakhoury M., Negrulj R., Mooranian A., Al-Salami H. (2014). Inflammatory bowel disease: Clinical aspects and treatments. J. Inflamm. Res..

[2] Kuenzig M.E., Fung S.G., Marderfeld L., Mak J.W.Y., Kaplan G.G., Ng S.C., Wilson D.C., Cameron F., Henderson P., Kotze P.G. (2022). Twenty-first century trends in the global epidemiology of pediatric-onset inflammatory bowel disease: Systematic review. Gastroenterology.

[3] Yu Y.R., Rodriguez J.R. (2017). Clinical presentation of Crohn’s, ulcerative colitis, and indeterminate colitis: Symptoms, extraintestinal manifestations, and disease phenotypes. Semin. Pediatr. Surg..

[4] D’Arcangelo G., Aloi M. (2017). Inflammatory bowel disease-unclassified in children: Diagnosis and pharmacological management. Paediatr. Drugs.

[5] Chandradevan R., Hofmekler T., Mondal K., Harun N., Venkateswaran S., Somineni H.K., Ballengee C.R., Kim M.-O., Griffiths A., Noe J.D. (2018). Evolution of pediatric inflammatory bowel disease unclassified (IBD-U): Incorporated with serological and gene expression profiles. Inflamm. Bowel Dis..

[6] Sýkora J., Pomahačová R., Kreslová M., Cvalínová D., Štych P., Schwarz J. (2018). Current global trends in the incidence of pediatric-onset inflammatory bowel disease. World J. Gastroenterol..

[7] Aardoom M.A., Veereman G., de Ridder L. (2019). A review on the use of anti-TNF in children and adolescents with inflammatory bowel disease. Int. J. Mol. Sci..

[8] Shah P., McDonald D. (2021). Vedolizumab: An emerging treatment option for pediatric inflammatory bowel disease. J. Pediatr. Pharmacol. Ther..

[9] Zhang W., Scalori A., Fuh F., McBride J., She G., Kierkus J., Korczowksi B., Li R., Abouhossein M., Kadva A. (2022). Pharmacokinetics, pharmacodynamics, and safety of Etrolizumab in children with moderately to severely active ulcerative colitis or crohn’s disease: Results from a phase 1 randomized trial. Inflamm. Bowel Dis..

[10] Okou D.T., Kugathasan S. (2014). Role of genetics in pediatric inflammatory bowel disease. Inflamm. Bowel Dis..

[11] Shouval D.S., Rufo P.A. (2017). The role of environmental factors in the pathogenesis of inflammatory bowel diseases: A review. JAMA Pediatr..

[12] Alsharairi N.A. (2020). The infant gut microbiota and risk of asthma: The effect of maternal nutrition during pregnancy and lactation. Microorganisms.

[13] Thursby E., Juge N. (2017). Introduction to the human gut microbiota. Biochem. J..

[14] Ríos-Covián D., Ruas-Madiedo P., Margolles A., Gueimonde M., de Los Reyes-Gavilán C.G., Salazar N. (2016). Intestinal short chain fatty acids and their link with diet and human health. Front. Microbiol..

[15] Den Besten G., van Eunen K., Groen A.K., Venema K., Reijngoud D., Bakker B.M. (2013). The role of short-chain fatty acids in the interplay between diet, gut microbiota, and host energy metabolism. J. Lipid Res..

[16] Fitzgerald R.S., Sanderson I.R., Claesson M.J. (2021). Paediatric inflammatory bowel disease and its relationship with the microbiome. Microb. Ecol..

[17] Zhuang X., Liu C., Zhan S., Tian Z., Li N., Mao R., Zeng Z., Chen M. (2021). Gut microbiota profile in pediatric patients with inflammatory bowel disease: A systematic review. Front. Pediatr..

[18] Sugihara K., Kamada N. (2021). Diet-microbiota interactions in inflammatory bowel disease. Nutrients.

[19] Healey G.R., Celiberto L.S., Lee S.M., Jacobson K. (2020). Fiber and prebiotic interventions in pediatric inflammatory bowel disease: What role does the gut microbiome play?. Nutrients.

[20] Olendzki B., Bucci V., Cawley C., Maserati R., McManus M., Olednzki E., Madziar C., Chiang D., Ward D.V., Pellish R. (2022). Dietary manipulation of the gut microbiome in inflammatory bowel disease patients: Pilot study. Gut Microbes.

[21] Levine A., Wine E., Assa A., Boneh R.S., Shaoul R., Kori M., Cohen S., Peleg S., Shamaly H., On A. (2019). Crohn’s disease exclusion diet plus partial enteral nutrition induces sustained remission in a randomized controlled trial. Gastroenterology.

[22] Boneh R.S., Van Limbergen J., Wine E., Assa A., Shaoul R., Milman P., Cohen S., Kori M., Peleg S., On A. (2021). Dietary therapies induce rapid response and remission in pediatric patients with active crohn’s disease. Clin. Gastroenterol. Hepatol..

[23] Scarallo L., Banci E., Pierattini V., Lionetti P. (2021). Crohn’s disease exclusion diet in children with Crohn’s disease: A case series. Curr. Med. Res. Opin..

[24] Matuszczyk M., Meglicka M., Landowski P., Czkwianianc E., Sordyl B., Szymańska E., Kierkuś J. (2021). Oral exclusive enteral nutrition for induction of clinical remission, mucosal healing, and improvement of nutritional status and growth velocity in children with active Crohn’s disease—A prospective multicentre trial. Prz. Gastroenterol..

[25] Niseteo T., Sila S., Trivić I., Mišak Z., Kolaček S., Hojsak I. (2022). Modified Crohn’s disease exclusion diet is equally effective as exclusive enteral nutrition: Real-world data. Nutr. Clin. Pract..

[26] MacLellan A., Moore-Connors J., Grant S., Cahill L., Langille M.G.I., Van Limbergen J. (2017). The Impact of exclusive enteral nutrition (EEN) on the gut microbiome in Crohn’s disease: A review. Nutrients.

[27] Diederen K., Li J.V., Donachie G.E., de Meij T.G., de Waart D.R., Hakvoort T.B.M., Kindermann A., Wagner J., Auyeung V., Te Velde A.A. (2020). Exclusive enteral nutrition mediates gut microbial and metabolic changes that are associated with remission in children with Crohn’s disease. Sci. Rep..

[28] Hart L., Verburgt C.M., Wine E., Zachos M., Poppen A., Chavannes M., Van Limbergen J., Pai N. (2021). Nutritional therapies and their influence on the intestinal microbiome in pediatric inflammatory bowel disease. Nutrients.

[29] Suskind D.L., Lee D., Kim Y.-M., Wahbeh G., Singh N., Braly K., Nuding M., Nicora C.D., Purvine S.O., Lipton M.S. (2020). The specific carbohydrate diet and diet modification as induction therapy for pediatric crohn’s disease: A randomized diet controlled trial. Nutrients.

[30] Shankar V., Gouda M., Moncivaiz J., Gordon A., Reo N.V., Hussein L., Paliy O. (2017). Differences in gut metabolites and microbial composition and functions between Egyptian and U.S. children are consistent with their diets. mSystems.

[31] El Amrousy D., Elashry H., Salamah A., Maher S., Abd-Elsalam A.M., Hasan S. (2022). Adherence to the mediterranean diet improved clinical scores and inflammatory markers in children with active inflammatory bowel disease: A randomized trial. J. Inflamm. Res..

[32] Alsharairi N.A. (2020). The role of short-chain fatty acids in the interplay between a very low-calorie ketogenic diet and the infant gut microbiota and its therapeutic implications for reducing asthma. Int. J. Mol. Sci..

[33] Alsharairi N.A. (2021). The role of short-chain fatty acids in mediating very low-calorie ketogenic diet-infant gut microbiota relationships and its therapeutic potential in obesity. Nutrients.

[34] Tóth C., Dabóczi A., Howard M., Miller N.J., Clemens Z. (2016). Crohn’s disease successfully treated with the paleolithic ketogenic diet. Int. J. Case Rep. Images.

[35] Loddo I., Romano C. (2015). Inflammatory bowel disease: Genetics, epigenetics, and pathogenesis. Front. Immunol..

[36] Li X., Song P., Timofeeva M., Meng X., Rudan I., Little J., Satsangi J., Campbell H., Theodoratou E. (2016). Systematic meta-analyses and field synopsis of genetic and epigenetic studies in paediatric inflammatory bowel disease. Sci. Rep..

[37] Fofanova T.Y., Petrosino J.F., Kellermayer R. (2016). Microbiome-epigenome interactions and the environmental origins of inflammatory bowel diseases. J. Pediatr. Gastroenterol. Nutr..

[38] Kelly D., Kotliar M., Woo V., Jagannathan S., Whitt J., Moncivaiz J., Aronow B.J., Dubinsky M.C., Hyams J.S., Markowitz J.F. (2018). Microbiota-sensitive epigenetic signature predicts inflammation in Crohn’s disease. JCI Insight.

[39] Harris R.A., Nagy-Szakal D., Mir S.A.V., Frank E., Szigeti R., Kaplan J.L., Bronsky J., Opekun A., Ferry G.D., Winter H. (2014). DNA methylation-associated colonic mucosal immune and defense responses in treatment-naïve pediatric ulcerative colitis. Epigenetics.

[40] McDermott E., Ryan E.J., Tosetto M., Gibson D., Burrage J., Keegan D., Byrne K., Crowe E., Sexton G., Malone K. (2016). DNA methylation profiling in inflammatory bowel disease provides new Insights into disease pathogenesis. J. Crohns Colitis.

[41] Harris R.A., Shah R., Hollister E.B., Tronstad R.R., Hovdenak N., Szigeti R., Versalovic J., Kellermayer R. (2016). Colonic mucosal epigenome and microbiome development in children and adolescents. J. Immunol. Res..

[42] Latronico A.C., Hochberg Z. (2010). G protein-coupled receptors in child development, growth, and maturation. Sci. Signal..

[43] Zenewicz L.A., Antov A., Flavell R.A. (2009). CD4 T-cell differentiation and inflammatory bowel disease. Trends Mol. Med..

[44] Fu S.-H., Chien M.-W., Hsu C.-Y., Liu Y.-W., Sytwu H.-K. (2020). Interplay between cytokine circuitry and transcriptional regulation shaping helper T cell pathogenicity and plasticity in inflammatory bowel disease. Int. J. Mol. Sci..

[45] Vasilyeva E., Abdulkhakov S., Cherepnev G., Martynova E., Mayanskaya I., Valeeva A., Abdulkhakov R., Safina D., Khaiboullina S., Rizvanov A. (2016). Serum cytokine profiles in children with crohn’s disease. Mediat. Inflamm..

[46] Mlakar A.S., Hojsak I., Jergović M., Čimić S., Bendelja K. (2018). Pediatric Crohn disease is characterized by Th1 in the terminal ileum and Th1/Th17 immune response in the colon. Eur. J. Pediatr..

[47] Ohtani K., Ohtsuka Y., Ikuse T., Baba Y., Yamakawa Y., Aoyagi Y., Fujii T., Kudo T., Nagata S., Shimizu T. (2010). Increased mucosal expression of GATA-3 and STAT-4 in pediatric ulcerative colitis. Pediatr. Int..

[48] Rosen M.J., Karns R., Vallance J.E., Bezold R., Waddell A., Collins M.H., Haberman Y., Minar P., Baldassano R.N., Hyams J.S. (2017). Mucosal expression of type 2 and type 17 immune response genes distinguishes ulcerative colitis from colon-only Crohn’s disease in treatment-naive pediatric patients. Gastroenterology.

[49] Cho J., Kim S., Yang D.H., Lee J., Park K.W., Go J., Hyun C.-L., Jee Y., Kang K.S. (2018). Mucosal immunity related to FOXP3^+^ regulatory T cells, Th17 cells and cytokines in pediatric inflammatory bowel disease. J. Korean Med. Sci..

[50] Sznurkowska K., Luty J., Bryl E., Witkowski J.M., Hermann-Okoniewska B., Landowski P., Kosek M., Szlagatys-Sidorkiewicz A. (2020). Enhancement of circulating and intestinal T regulatory cells and their expression of helios and neuropilin-1 in children with inflammatory bowel disease. J. Inflamm. Res..

[51] Reikvam D.H., Perminow G., Lyckander L.G., Gran J.M., Brandtzaeg P., Vatn M., Carlsen H.S. (2011). Increase of regulatory T cells in ileal mucosa of untreated pediatric Crohn’s disease patients. Scand. J. Gastroenterol..

[52] Elshal M.F., Aldahlawi A.M., Saadah O.I., McCoy J.P. (2015). Reduced Dendritic Cells Expressing CD200R1 in Children with Inflammatory Bowel Disease: Correlation with Th17 and Regulatory T Cells. Int. J. Mol. Sci..

[53] Zhu X.-M., Shi Y.Z., Cheng M., Wang D.F., Fan J.F. (2017). Serum IL-6, IL-23 profile and Treg/Th17 peripheral cell populations in pediatric patients with inflammatory bowel disease. Pharmazie.

[54] Ricciardelli I., Lindley K.J., Londei M., Quaratino S. (2008). Anti-tumour necrosis-α therapy increases the number of FOXP3^+^ regulatory T cells in children affected by Crohn’s disease. Immunology.

[55] Vitale A., Strisciuglio C., Vitale S., Santopaolo M., Bruzzese D., Micillo T., Scarpato E., Miele E., Staiano A., Troncone R. (2020). Increased frequency of regulatory T cells in pediatric inflammatory bowel disease at diagnosis: A compensative role?. Pediatr. Res..

[56] Ruiz Castro P.A., Yepiskoposyan H., Gubian S., Calvino-Martin F., Kogel U., Renggli K., Peitsch M.C., Hoeng J., Talikka M. (2021). Systems biology approach highlights mechanistic differences between Crohn’s disease and ulcerative colitis. Sci. Rep..

[57] Bassett S.A., Barnett M.P.G. (2014). The role of dietary histone deacetylases (HDACs) inhibitors in health and disease. Nutrients.

[58] Mokry M., Middendorp S., Wiegerinck C.L., Witte M., Teunissen H., Meddens C.A., Cuppen E., Clevers H., Nieuwenhuis E.E.S. (2014). Many inflammatory bowel disease risk loci include regions that regulate gene expression in immune cells and the intestinal epithelium. Gastroenterology.

[59] Felice C., Lewis A., Iqbal C., Gordon H., Rigoni A., Colombo M.P., Armuzzi A., Feakins R., Lindsay J.O., Silver A. (2021). Intestinal inflammation is linked to hypoacetylation of histone 3 lysine 27 and can be reversed by valproic acid treatment in inflammatory bowel disease patients. Cell. Mol. Gastroenterol. Hepatol..

[60] Li C., Chen Y., Zhu H., Zhang X., Han L., Zhao Z., Wang J., Ning L., Zhou W., Lu C. (2020). Inhibition of histone deacetylation by MS-275 alleviates colitis by activating the vitamin D receptor. J. Crohns Colitis.

[61] Stronati L., Negroni A., Merola P., Pannone V., Borrelli O., Cirulli M., Annese V., Cucchiara S. (2008). Mucosal NOD2 expression and NF-kappaB activation in pediatric Crohn’s disease. Inflamm. Bowel Dis..

[62] Shilpa J., Mohan V. (2018). Ketogenic diets: Boon or bane?. Indian J. Med. Res..

[63] Nall R. (2018). What Foods Should You Eat on a Ketogenic Diet. Medical News Today.

[64] Knudsen K.A.B. (2015). Microbial degradation of whole-grain complex carbohydrates and impact on short-chain fatty acids and health. Adv. Nutr..

[65] Morrison D.J., Preston T. (2016). Formation of short chain fatty acids by the gut microbiota and their impact on human metabolism. Gut Microbes.

[66] Holscher H.D. (2017). Dietary fiber and prebiotics and the gastrointestinal microbiota. Gut Microbes.

[67] Ahmadi S., Mainali R., Nagpal R., Sheikh-Zeinoddin M., Soleimanian-Zad S., Wang S., Deep G., Mishra S.K., Yadav H. (2017). Dietary polysaccharides in the amelioration of gut microbiome dysbiosis and metabolic diseases. Obes. Control Ther..

[68] Ludwig D.S., Hu F.B., Tappy L., Brand-Miller J. (2018). Dietary carbohydrates: Role of quality and quantity in chronic disease. BMJ.

[69] Zhang T., Yang Y., Liang Y., Jiao X., Zhao C. (2018). Beneficial effect of intestinal fermentation of natural polysaccharides. Nutrients.

[70] Dhillon K.K., Gupta S. (2022). Biochemistry, Ketogenesis.

[71] Newman J.C., Verdin E. (2014). β-hydroxybutyrate: Much more than a metabolite. Diabetes Res. Clin. Pract..

[72] Cavaleri F., Bashar E. (2018). Potential synergies of β-hydroxybutyrate and butyrate on the modulation of metabolism, inflammation, cognition, and general health. J. Nutr. Metab..

[73] Sasaki K., Sasaki D., Hannya A. (2020). In vitro human colonic microbiota utilises D-β-hydroxybutyrate to increase butyrogenesis. Sci. Rep..

[74] Aleksandrova K., Romero-Mosquera B., Hernandez V. (2017). Diet, gut microbiome and epigenetics: Emerging links with inflammatory bowel diseases and prospects for management and prevention. Nutrients.

[75] Dolan K.T., Chang E.B. (2017). Diet, gut microbes, and the pathogenesis of inflammatory bowel diseases. Mol. Nutr. Food Res..

[76] Zhang Z., Zhang H., Chen T., Shi L., Wang D., Tang D. (2022). Regulatory role of short-chain fatty acids in inflammatory bowel disease. Cell Commun. Signal..

[77] Sun M., Wu W., Liu Z., Cong Y. (2017). Microbiota metabolite short chain fatty acids, GPCR, and inflammatory bowel diseases. J. Gastroenterol..

[78] Deleu S., Machiels K., Raes J., Verbeke K., Vermeire S. (2021). Short chain fatty acids and its producing organisms: An overlooked therapy for IBD?. EBioMedicine.

[79] Xu H.-M., Zhao H.-L., Guo G.J., Xu J., Zhou Y.-L., Huang H.-L., Nie Y.-Q. (2022). Characterization of short-chain fatty acids in patients with ulcerative colitis: A meta-analysis. BMC Gastroenterol..

[80] Zhou Z., Cao J., Liu X., Li M. (2021). Evidence for the butyrate metabolism as key pathway improving ulcerative colitis in both pediatric and adult patients. Bioengineered.

[81] Jurickova I., Bonkowski E., Angerman E., Novak E., Huron A., Akers G., Iwasawa K., Braun T., Hadar R., Hooker M. (2022). Eicosatetraynoic acid and butyrate regulate human intestinal organoid mitochondrial and extracellular matrix pathways implicated in Crohn’s disease strictures. Inflamm. Bowel Dis..

[82] Pace F., Rudolph S.E., Chen Y., Bao B., Kaplan D.L., Watnick P.I. (2021). The short-chain fatty acids propionate and butyrate augment adherent-invasive *Escherichia coli* virulence but repress inflammation in a human intestinal enteroid model of infection. Microbiol. Spectr..

[83] Segain J., de la Bletiere D.R., Bourreille A., Leray V., Gervois N., Rosales C., Ferrier L., Bonnet C., Blottiere H., Galmiche J. (2000). Butyrate inhibits inflammatory responses through NFκB inhibition: Implications for Crohn’s disease. Gut.

[84] Tedelind S., Westberg F., Kjerrulf M., Vidal A. (2007). Anti-inflammatory properties of the short-chain fatty acids acetate and propionate: A study with relevance to inflammatory bowel disease. World J. Gastroenterol..

[85] Agus A., Denizot J., Thévenot J., Martinez-Medina M., Massier S., Sauvanet P., Bernalier-Donadille A., Denis S., Hofman P., Bonnet R. (2016). Western diet induces a shift in microbiota composition enhancing susceptibility to Adherent-Invasive *E. coli* infection and intestinal inflammation. Sci. Rep..

[86] D’Souza W.N., Douangpanya J., Mu S., Jaeckel P., Zhang M., Maxwell J.R., Rottman J.B., Labitzke K., Willee A., Beckmann H. (2017). Differing roles for short chain fatty acids and GPR43 agonism in the regulation of intestinal barrier function and immune responses. PLoS ONE.

[87] Hamer H.M., Jonkers D., Venema K., Vanhoutvin S., Troost F.J., Brummer R.-J. (2008). Review article: The role of butyrate on colonic function. Aliment. Pharmacol. Ther..

[88] Gerbeth L., Glauben R. (2021). Histone deacetylases in the inflamed intestinal epithelium-promises of new therapeutic strategies. Front. Med..

[89] Venegas D.P., De la Fuente M.K., Landskron G., González M.J., Quera R., Dijkstra G., Harmsen H.J.M., Faber K.N., Hermoso M.A. (2019). Short chain fatty acids (SCFAs)-mediated gut epithelial and immune regulation and its relevance for inflammatory bowel diseases. Front. Immunol..

[90] Huang C., Wang J., Liu H., Huang R., Yan X., Song M., Tan G., Zhi F. (2022). Ketone body β-hydroxybutyrate ameliorates colitis by promoting M2 macrophage polarization through the STAT6-dependent signaling pathway. BMC Med..

[91] Dąbek A., Wojtala M., Pirola L., Balcerczyk A. (2020). Modulation of cellular biochemistry, epigenetics and metabolomics by ketone bodies. Implications of the ketogenic diet in the physiology of the organism and pathological states. Nutrients.

[92] Zhou L., Liu T., Huang B., Luo M., Chen Z., Zhao Z., Wang J., Leung D., Yang X., Chan K.W. (2021). Excessive deubiquitination of NLRP3-R779C variant contributes to very-early-onset inflammatory bowel disease development. J. Allergy Clin. Immunol..

[93] Youm Y.-H., Nguyen K.Y., Grant R.W., Goldberg E.L., Bodogai M., Kim D., D’Agostino D., Planavsky N., Lupfer C., Kanneganti T.D. (2015). The ketone metabolite β-hydroxybutyrate blocks NLRP3 inflammasome-mediated inflammatory disease. Nat. Med..

[94] Huang H., Zhang D., Weng Y., Delaney K., Tang Z., Yan C., Qi S., Peng C., Cole P.A., Roeder R.G. (2021). The regulatory enzymes and protein substrates for the lysine β-hydroxybutyrylation pathway. Sci. Adv..

[95] O’Neill I., Schofield Z., Hall L.J. (2017). Exploring the role of the microbiota member *Bifidobacterium* in modulating immune-linked diseases. Emerg. Top. Life Sci..

[96] Celiberto L.S., Graef F.A., Healey G.R., Bosman E.S., Jacobson K., Sly L.M., Vallance B.A. (2018). Inflammatory bowel disease and immunonutrition: Novel therapeutic approaches through modulation of diet and the gut microbiome. Immunology.

[97] Jakubczyk D., Leszczyńska K., Górska S. (2020). The Effectiveness of probiotics in the treatment of inflammatory bowel disease (IBD)—A critical review. Nutrients.

[98] Darb Emamie A., Rajabpour M., Ghanavati R., Asadolahi P., Farzi S., Sobouti B., Darbandi A. (2021). The effects of probiotics, prebiotics and synbiotics on the reduction of IBD complications, a periodic review during 2009–2020. J. Appl. Microbiol..

[99] Yao S., Zhao Z., Wang W., Liu X. (2021). Bifidobacterium Longum: Protection against inflammatory bowel disease. J. Immunol. Res..

[100] Le B., Yang S.H. (2018). Efficacy of *Lactobacillus plantarum* in prevention of inflammatory bowel disease. Toxicol. Rep..

[101] Sichetti M., De Marco S., Pagiotti R., Traina G., Pietrella D. (2018). Anti-inflammatory effect of multistrain probiotic formulation (*L. rhamnosus*, *B. lactis*, and *B. longum*). Nutrition.

[102] Leccese G., Bibi A., Mazza S., Facciotti F., Caprioli F., Landini P., Paroni M. (2020). Probiotic lactobacillus and bifidobacterium strains counteract adherent-invasive *Escherichia coli* (AIEC) virulence and hamper IL-23/Th17 axis in ulcerative colitis, but not in crohn’s disease. Cells.

[103] Riedel C.U., Foata F., Philippe D., Adolfsson O., Eikmanns B.J., Blum S. (2006). Anti-inflammatory effects of bifidobacteria by inhibition of LPS-induced NF-κB activation. World J. Gastroenterol..

[104] Hsieh C.-Y., Osaka T., Moriyama E., Date Y., Kikuchi J., Tsuneda S. (2015). Strengthening of the intestinal epithelial tight junction by *Bifidobacterium bifidum*. Physiol. Rep..

[105] Imaoka A., Shima T., Kato K., Mizuno S., Uehara T., Matsumoto S., Setoyama H., Hara T., Umesaki Y. (2008). Anti-inflammatory activity of probiotic *Bifidobacterium*: Enhancement of IL-10 production in peripheral blood mononuclear cells from ulcerative colitis patients and inhibition of IL-8 secretion in HT-29 cells. World J. Gastroenterol..

[106] O’Hara A.M., O’Regan P., Fanning A., O’Mahony C., Macsharry J., Lyons A., Bienenstock J., O’Mahony L., Shanahan F. (2006). Functional modulation of human intestinal epithelial cell responses by *Bifidobacterium infantis* and *Lactobacillus salivarius*. Immunology.

[107] Kang S., Lin Z., Xu Y., Park M., Ji G.E., Johnston T.V., Ku S., Park M.S. (2022). A recombinant *Bifidobacterium bifidum* BGN4 strain expressing the streptococcal superoxide dismutase gene ameliorates inflammatory bowel disease. Microb. Cell Fact..

[108] Kanmani P., Kim H. (2020). Beneficial effect of immunobiotic strains on attenuation of Salmonella induced inflammatory response in human intestinal epithelial cells. PLoS ONE.

[109] Hernández-Delgado N.C., Torres-Maravilla E., Mayorga-Reyes L., Martín R., Langella P., Pérez-Pastén-Borja R., Sánchez-Pardo M.E., Bermúdez-Humarán L.G. (2021). Antioxidant and anti-inflammatory properties of probiotic candidate strains isolated during fermentation of Agave (*Agave angustifolia* Haw). Microorganisms.

[110] Curciarello R., Canziani K.E., Salto I., Romero E.B., Rocca A., Doldan I., Peton E., Brayer S., Sambuelli A.M., Goncalves S. (2021). Probiotic Lactobacilli isolated from Kefir promote down-regulation of inflammatory lamina propria T cells from patients with active IBD. Front. Pharmacol..

[111] Okada Y., Tsuzuki Y., Hokari R., Komoto S., Kurihara C., Kawaguchi A., Nagao S., Miura S. (2009). Anti-inflammatory effects of the genus *Bifidobacterium* on macrophages by modification of phospho-IκB and SOCS gene expression. Int. J. Exp. Pathol..

[112] Olivares M., Castillejo G., Varea V., Sanz Y. (2014). Double-blind, randomised, placebo-controlled intervention trial to evaluate the effects of Bifidobacterium longum CECT 7347 in children with newly diagnosed coeliac disease. Br. J. Nutr..

[113] Oliva S., Di Nardo G., Ferrari F., Mallardo S., Rossi P., Patrizi G., Cucchiara S., Stronati L. (2012). Randomised clinical trial: The effectiveness of *Lactobacillus reuteri* ATCC 55730 rectal enema in children with active distal ulcerative colitis. Aliment. Pharmacol. Ther..

[114] Scott K.P., Martin J.C., Duncan S.H., Flint H.J. (2014). Prebiotic stimulation of human colonic butyrate-producing bacteria and bifidobacteria, in vitro. FEMS Microbiol. Ecol..

[115] Chia L.W., Mank M., Blijenberg B., Aalvink S., Bongers R.S., Stahl B., Knol J., Belzer C. (2020). *Bacteroides thetaiotaomicron* fosters the growth of butyrate-producing *Anaerostipes caccae* in the presence of lactose and total human milk carbohydrates. Microorganisms.

[116] Conte M.P., Schippa S., Zamboni I., Penta M., Chiarini F., Seganti L., Osborn J., Falconieri P., Borrelli O., Cucchiara S. (2006). Gut-associated bacterial microbiota in paediatric patients with inflammatory bowel disease. Gut.

[117] Hansen R., Sanderson I.R., Muhammed R., Allen S., Tzivinikos C., Henderson P., Gervais L., Jeffery I.B., Mullins D.P., O’Herlihy E.A. (2020). A Double-blind, placebo-controlled trial to assess safety and tolerability of (Thetanix) Bacteroides thetaiotaomicron in adolescent crohn’s disease. Clin. Transl. Gastroenterol..

[118] Haller D., Holt L., Parlesak A., Zanga J., Bäuerlein A., Sartor R.B., Jobin C. (2004). Differential effect of immune cells on non-pathogenic Gram-negative bacteria-induced nuclear factor-κB activation and pro-inflammatory gene expression in intestinal epithelial cells. Immunology.

[119] Di Lorenzo F., Pither M.D., Martufi M., Scarinci I., Guzmán-Caldentey J., Łakomiec E., Jachymek W., Bruijns S.C.M., Santamaría S.M., Frick J. (2020). Pairing *Bacteroides vulgatus* LPS structure with its immunomodulatory e_ects on human cellular models. ACS Cent. Sci..

[120] Durant L., Stentz R., Noble A., Brooks J., Gicheva N., Reddi D., O’Connor M.J., Hoyles L., McCartney A.L., Man R. (2020). Bacteroides thetaiotaomicron-derived outer membrane vesicles promote regulatory dendritic cell responses in health but not in inflammatory bowel disease. Microbiome.

[121] Li K., Hao Z., Du J., Gao Y., Yang S., Zhou Y. (2021). Bacteroides thetaiotaomicron relieves colon inflammation by activating aryl hydrocarbon receptor and modulating CD4^+^ T cell homeostasis. Int. Immunopharmacol..

[122] Zhang L., Liu F., Xue J., Lee S.A., Liu L., Riordan S.M. (2022). Bacterial species associated with human inflammatory bowel disease and their pathogenic mechanisms. Front. Microbiol..

[123] Zitomersky N.L., Atkinson B.J., Franklin S.W., Mitchell P.D., Snapper S.B., Comstock L.E., Bousvaros A. (2013). Characterization of adherent bacteroidales from intestinal biopsies of children and young adults with inflammatory bowel disease. PLoS ONE.

[124] Telesford K.M., Yan W., Ochoa-Reparaz J., Pant A., Kircher C., Christy M.A., Begum-Haque S., Kasper D.L., Kasper L.H. (2015). A commensal symbiotic factor derived from *Bacteroides fragilis* promotes human CD39^+^Foxp3^+^ T cells and Treg function. Gut Microbes.

[125] Jiang F., Meng D., Weng M., Zhu W., Wu W., Kasper D., Walker W.A. (2017). The symbiotic bacterial surface factor polysaccharide A on *Bacteroides fragilis* inhibits IL-1β-induced inflammation in human fetal enterocytes via toll receptors 2 and 4. PLoS ONE.

[126] Becker H.E.F., Jamin C., Bervoets L., Boleij A., Xu P., Pierik M.J., Stassen F.R.M., Savelkoul P.H.M., Penders J., Jonkers D.M.A.E. (2021). Higher prevalence of *Bacteroides fragilis* in crohn’s disease exacerbations and strain-dependent increase of epithelial resistance. Front. Microbiol..

[127] Lenoir M., Martín R., Torres-Maravilla E., Chadi S., González-Dávila P., Sokol H., Langella P., Chain F., Bermúdez-Humarán L.G. (2020). Butyrate mediates anti-inflammatory effects of *Faecalibacterium prausnitzii* in intestinal epithelial cells through *Dact3*. Gut Microbes.

[128] Schwiertz A., Jacobi M., Frick J.-S., Richter M., Rusch K., Köhler H. (2010). Microbiota in pediatric inflammatory bowel disease. J. Pediatr..

[129] Kowalska-Duplaga K., Gosiewski T., Kapusta P., Sroka-Oleksiak A., Wędrychowicz A., Pieczarkowski S., Ludwig-Słomczyńska A.H., Wołkow P.P., Fyderek K. (2019). Differences in the intestinal microbiome of healthy children and patients with newly diagnosed Crohn’s disease. Sci. Rep..

[130] Sokol H., Pigneur B., Watterlot L., Lakhdari O., Bermúdez-Humarán L.G., Gratadoux J.-J., Blugeon S., Bridonneau C., Furet J.-P., Corthier G. (2008). Faecalibacterium prausnitzii is an anti-inflammatory commensal bacterium identified by gut microbiota analysis of Crohn disease patients. Proc. Natl. Acad. Sci. USA.

[131] Rossi O., van Berkel L.A., Chain F., Khan M.T., Taverne N., Sokol H., Duncan S.H., Flint H.J., Harmsen H.J.M., Langella P. (2016). *Faecalibacterium prausnitzii* A2-165 has a high capacity to induce IL-10 in human and murine dendritic cells and modulates T cell responses. Sci. Rep..

[132] Martín R., Bermúdez-Humarán L.G., Langella P. (2018). Searching for the Bacterial Effector: The Example of the Multi-Skilled Commensal Bacterium *Faecalibacterium prausnitzii*. Front. Microbiol..

[133] Ulluwishewa D., Anderson R.C., Young W., McNabb W.C., van Baarlen P., Moughan P.J., Wells J.M., Roy N.C. (2015). Live Faecalibacterium prausnitzii in an apical anaerobic model of the intestinal epithelial barrier. Cell. Microbiol..

[134] Alameddine J., Godefroy E., Papargyris L., Sarrabayrouse G., Tabiasco J., Bridonneau C., Yazdanbakhsh K., Sokol H., Altare F., Jotereau F. (2019). *Faecalibacterium prausnitzii* skews human DC to prime IL10-producing T cells through TLR2/6/JNK signaling and IL-10, IL-27, CD39, and IDO-1 induction. Front. Immunol..

[135] Shah R., Cope J.L., Nagy-Szakal D., Dowd S., Versalovic D., Hollister E.B., Kellermayer R. (2016). Composition and function of the pediatric colonic mucosal microbiome in untreated patients with ulcerative colitis. Gut Microbes.

[136] El Mouzan M.I., Winter H.S., Assiri A.A., Korolev K.S., Al Sarkhy A.A., Dowd S.E., Al Mofarreh M.A., Menon R. (2018). Microbiota profile in new-onset pediatric Crohn’s disease: Data from a non-Western population. Gut Pathog..

[137] Nie K., Ma K., Luo W., Shen Z., Yang Z., Xiao M., Tong T., Yang Y., Wang X. (2021). *Roseburia intestinalis*: A beneficial gut organism from the discoveries in genus and species. Front. Cell. Infect. Microbiol..

[138] Olbjørn C., Småstuen M.C., Moen A.E.F. (2022). Targeted analysis of the gut microbiome for diagnosis, prognosis and treatment individualization in pediatric inflammatory bowel disease. Microorganisms.

[139] Falony G., Vlachou A., Verbrugghe K., De Vuyst L. (2006). Cross-feeding between *Bifidobacterium longum* BB536 and acetate-converting, butyrate-producing colon bacteria during growth on oligofructose. Appl. Environ. Microbiol..

[140] De Vuyst L., Leroy F. (2011). Cross-feeding between bifidobacteria and butyrate-producing colon bacteria explains bifdobacterial competitiveness, butyrate production, and gas production. Int. J. Food Microbiol..

[141] Zhu C., Song K., Shen Z., Quan Y., Tan B., Luo W., Wu S., Tang K., Yang Z., Wang X. (2018). *Roseburia intestinalis* inhibits interleukin-17 excretion and promotes regulatory T cells differentiation in colitis. Mol. Med. Rep..

[142] Shen Z., Zhu C., Quan Y., Yang J., Yuan W., Yang Z., Wu S., Luo W., Tan B., Wang X. (2018). Insights into *Roseburia intestinalis* which alleviates experimental colitis pathology by inducing anti-inflammatory responses. J. Gastroenterol. Hepatol..

[143] Mohebali N., Ekat K., Kreikemeyer B., Breitrück A. (2020). Barrier protection and recovery effects of gut commensal bacteria on differentiated intestinal epithelial cells in vitro. Nutrients.

